# First application of a 6.3 Fr flexible ureteroscope in the treatment of bilateral staghorn calculi: A case report

**DOI:** 10.1097/MD.0000000000043524

**Published:** 2025-07-25

**Authors:** Feihong Xu, Rongfang Zhong, Weiyong Zhong, Shujiang Ye, Zhenquan Lu, Lin Xiong, Xiang Xu

**Affiliations:** a Department of Urology, The University of Hong Kong–Shenzhen Hospital, Shenzhen, Guangdong Province, China; b Hospital Management Committee of Shenzhen University, Shenzhen, Guangdong Province, China.

**Keywords:** flexible ureteroscopy, ratio of endoscope-to-sheath diameter, staghorn calculi

## Abstract

**Rationale:**

Staghorn calculi are a challenging type of kidney stone traditionally treated with percutaneous nephrolithotomy (PCNL) or multiple endoscopic procedures. However, these methods are often associated with a higher risk of postoperative complications and prolonged recovery periods. Recent advancements in flexible ureteroscopy (fURS) technology have offered the potential for improving the management of kidney stones, but its application in treating complex staghorn stones remains limited due to technical and anatomical challenges.

**Patient concerns:**

A patient presented with bilateral staghorn calculi, which posed significant challenges for treatment due to their size and complexity. The patient was concerned about the risks associated with the traditional methods, including postoperative infection, long recovery times, and potential complications.

**Diagnoses:**

The patient was diagnosed with bilateral staghorn calculi, large and complex stones that require a highly specialized approach for effective treatment.

**Interventions:**

A novel approach was used to manage the patient’s bilateral staghorn calculi using a 6.3 Fr flexible ureteroscope. Complete stone clearance was achieved using 2 procedures. The smaller sheath diameter of the 6.3 Fr flexible ureteroscope provides several advantages, including improved lithotripsy efficiency, reduced intrarenal pressure, and enhanced irrigation flow.

**Outcomes:**

Use of the 6.3 Fr flexible ureteroscope resulted in complete stone clearance with minimal postoperative complications, such as infection. This procedure demonstrated high efficiency and precision, highlighting the potential of advanced fURS technology for the treatment of complex kidney stones.

**Lessons:**

This case highlights the advantages of utilizing the 6.3 Fr flexible ureteroscope in the management of complex kidney stones, particularly staghorn calculi. These findings suggest a higher single-stage surgical success rate and fewer postoperative complications. This approach reflects the evolving field of endourology and demonstrates the potential for broader applications of the advanced fURS technology.

## 1. Introduction

Staghorn calculi are a complex type of kidney stone that occupy the renal pelvis and calyces and are often associated with risks of recurrent infections and progressive renal function impairment.^[1]^ The primary objective of treatment is to achieve complete stone clearance to preserve renal function and prevent long-term complications.^[2]^ Historically, standard treatments for staghorn calculi include percutaneous nephrolithotomy (PCNL), a combination of PCNL and flexible ureteroscopy (fURS), and, in rare cases, open surgery. Currently, PCNL remains the first-line treatment for staghorn calculi, while clinical guidelines recommend fURS primarily for stones smaller than 2 cm.^[3,4]^ Although PCNL offers superior stone clearance rates for large-volume stones, it is associated with a higher risk of complications, including significant bleeding, infection, thoracic injuries, and potential kidney damage.^[5]^

fURS, a minimally invasive treatment option, has become increasingly prominent in the management of kidney stones. Although the stone clearance rate of fURS for renal stones larger than 2 cm remains lower compared with that of PCNL, the advanced active and passive deflection capabilities of the flexible ureteroscope tip enable access to various renal calyces. This allows for precise navigation of complex calyceal structures and facilitates comprehensive examination of the renal collecting system, thereby minimizing the renal parenchymal trauma commonly associated with PCNL.^[6,7]^ In this case, we innovatively utilized a 6.3 Fr flexible ureteroscope. Compared with conventional flexible ureteroscope, which are typically 7.5 Fr or larger, the smaller diameter of the 6.3 Fr flexible ureteroscope reduces the sheath-to-scope ratio, thereby enhancing the intraoperative irrigation efficiency and lowering the intrarenal pressure. This provides several advantages, including reduced damage to the ureter and renal calyces, decreased risk of postoperative infection, and significantly improved maneuverability. These features make the 6.3 Fr flexible ureteroscope a promising tool for managing complex stones. This case report evaluates the clinical application of a 6.3 Fr flexible ureteroscope in the treatment of bilateral staghorn calculi, focusing on its advantages and potential role in the minimally invasive management of complex renal stones.

## 2. Case presentation

### 2.1. Patient information

Our patient is a 52-year-old Chinese female who began experiencing bilateral intermittent dull flank pain 3 years ago, with the right side being more pronounced. The pain was unrelated to any obvious triggers, improved with rest, and was not accompanied by fever, hematuria, urinary frequency, or urgency. The patient did not seek treatment at the time. Two weeks prior, she had developed intermittent hematuria, presenting as light red gross hematuria, without urinary frequency, urgency, dysuria, fever, or chills. She sought medical attention at a hospital in Hong Kong, where a computed tomography (CT) scan of the urinary system revealed bilateral staghorn calculi with stones measuring approximately 8 cm on the right side and 6 cm on the left side. A preoperative CT report in Hong Kong indicates that the HU value of the stone was approximately 650. The patient underwent atrial septal defect closure surgery in 2022 for congenital heart condition and was on regular clopidogrel therapy postoperatively. Clopidogrel was discontinued 1 week before admission.

### 2.2. Physical examination

There was no bulging in either renal region, and no tenderness was noted at the costovertebral angles on either side. Percussion pain was not observed in the renal areas. The abdomen was flat with soft abdominal muscles. Tenderness was not detected at the subcostal points, upper ureteral, or mid-ureteral points. The suprapubic bladder region showed no bulging, and percussion revealed dullness.

#### 2.2.1. X-ray

**Figure 1.**
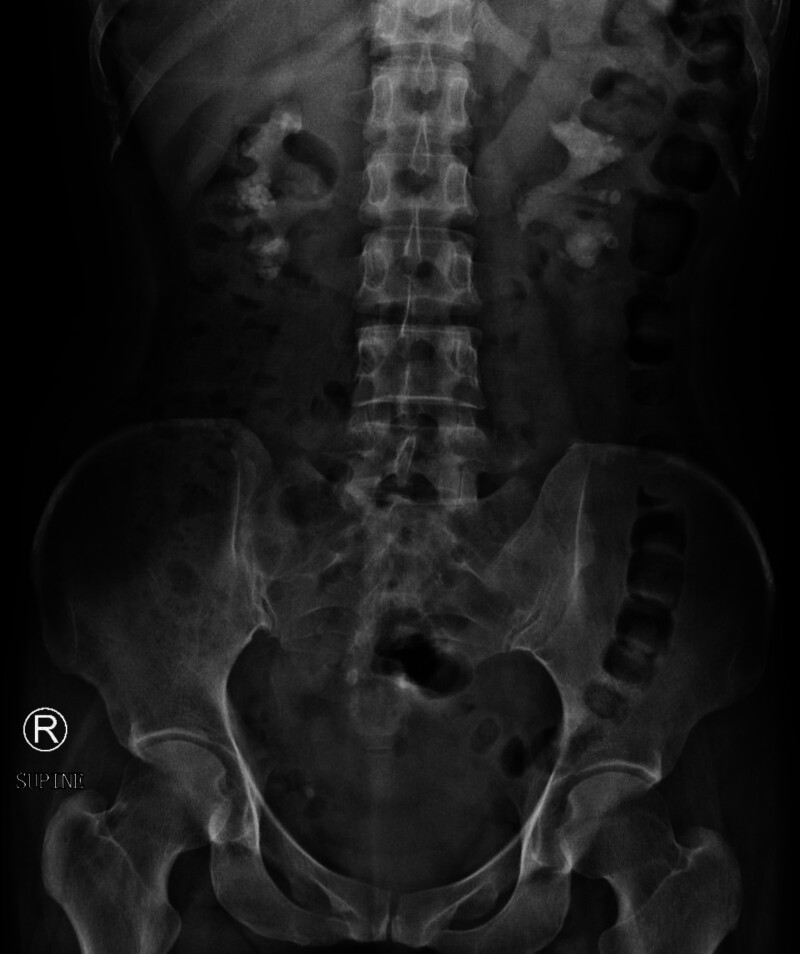
The visualization of both kidneys and psoas muscles is suboptimal. Cast-like and multiple granular high-density lesions are observed in the bilateral renal regions, with the largest measuring approximately 78 × 55 mm on the right and 67 × 46 mm on the left.

Multiple stones in both kidneys, left side measuring approximately 67 × 46 mm, right side measuring approximately 78 × 55 mm (Fig. 1).

#### 2.2.2. Laboratory tests

Preoperative blood test and liver and kidney function tests showed no significant abnormalities. Urine culture was negative on admission, but urinalysis revealed leukocytes, suggesting a urinary tract infection. Given the large stone burden, we administered a 3-day preoperative course of amoxicillin-clavulanate as antibiotic prophylaxis.

### 2.3. Treatment

We discuss the conditions and surgical options for the patient in detail. Owing to her history of anticoagulant use and concerns about renal parenchymal damage and bleeding, the patient opted for fURS for stone fragmentation.

#### 2.3.1. First surgery

**Figure 2.**
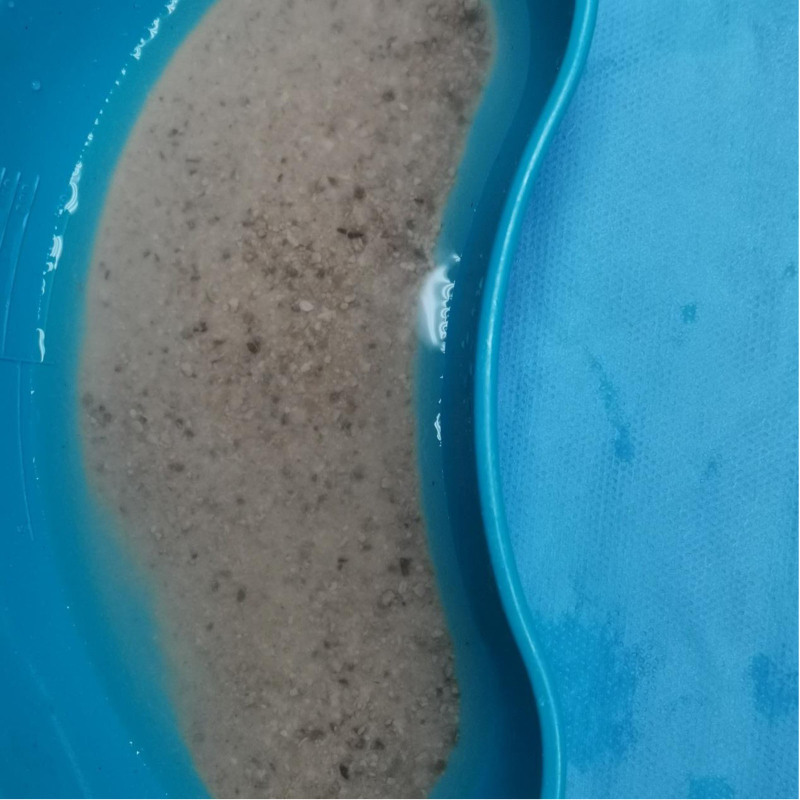
Intraoperative image of the calculus from the first surgery.

**Figure 3.**
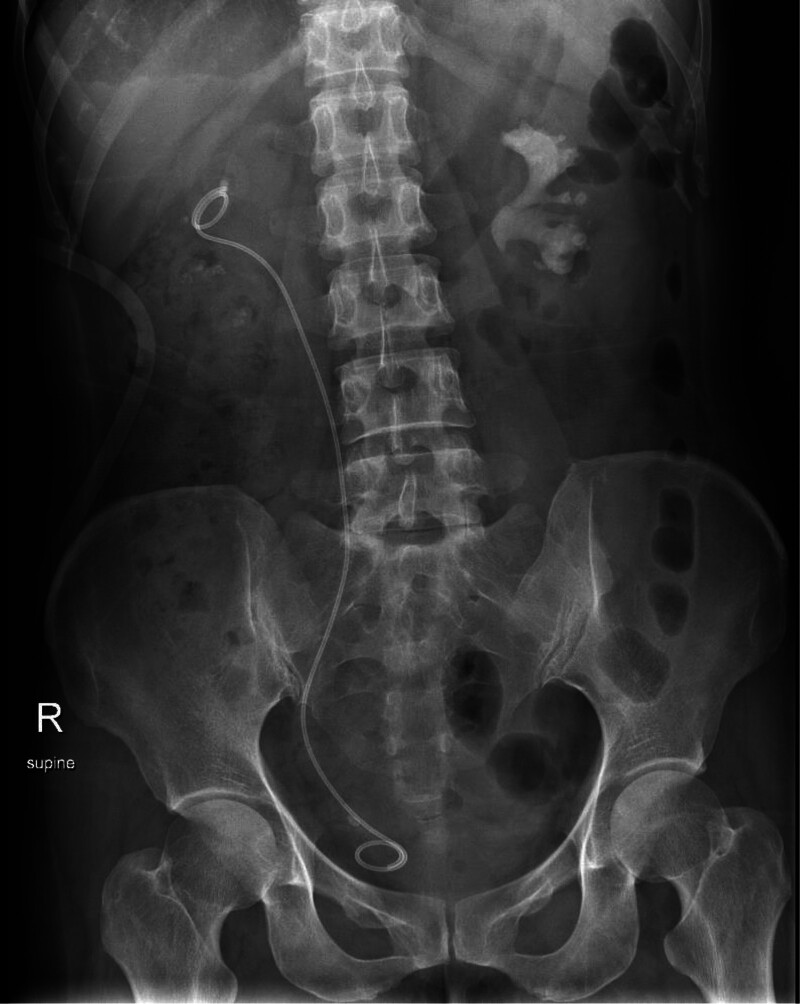
After the first surgery, an abdominal X-ray showed a significant reduction in the right-sided stones compared with before, and the right ureteral stent was in place.

Under general anesthesia, the patient was placed in the lithotomy position. A 6.3 Fr disposable flexible ureteroscope was inserted into the bladder through the urethral meatus under direct vision. The urethra and bladder were normal, and the ureteral orifices on both sides were clearly visible and slit-like. A 0.035 guidewire (150 cm; NiCore® Nitinol, Bard) was placed into the right ureter under direct vision. The guidewire was left in place, while the disposable flexible ureteroscope was removed. A 12 Fr flexible ureteral access sheath (TF-UAS; Shenzhen Kangyibo Technology Development Co., Ltd., Shenzhen, China) was introduced over the guidewire, followed by the reinsertion of the disposable flexible ureteroscope through the sheath. Upon entering the renal pelvis, right staghorn calculus, consistent with the preoperative CT size, was observed. A holmium laser fiber (Lumenis Pulse™ 100H Holmium Laser System, Boston Scientific, Natick ) was connected, and the stones were fragmented. Laser lithotripsy during retrograde intrarenal surgery was performed using a 200-µm laser fiber in dusting mode to optimize stone fragmentation and achieve a high stone-free rate. The energy and frequency settings were maintained at 0.4 to 0.6 J and 50 Hz. Most stone fragments within the visual field were evacuated using a negative pressure sheath (Fig. 2). A guidewire was inserted along with an F5 Marflow double-J stent (APR Medtech, Oxfordshire, UK). An F16 Foley catheter was placed. The procedure was completed successfully with an estimated intraoperative blood loss of approximately 2 mL. The surgery duration was maintained within 2 hours, according to the clinical guidelines. Second-stage surgery was planned for the residual small fragments on the right side and left kidney stones. Postoperative X-ray imaging showed a significant reduction in the size of the right kidney stones compared with the preoperative findings (Fig. 3). The first day after the first surgery, the patient’s white blood cell was 11.46 × 10^9^/L, interleukin-6 was 15.8 pg/mL, and C-reactive protein levels showed no significant abnormality in kidney function.

#### 2.3.2. Second surgery

**Figure 4.**
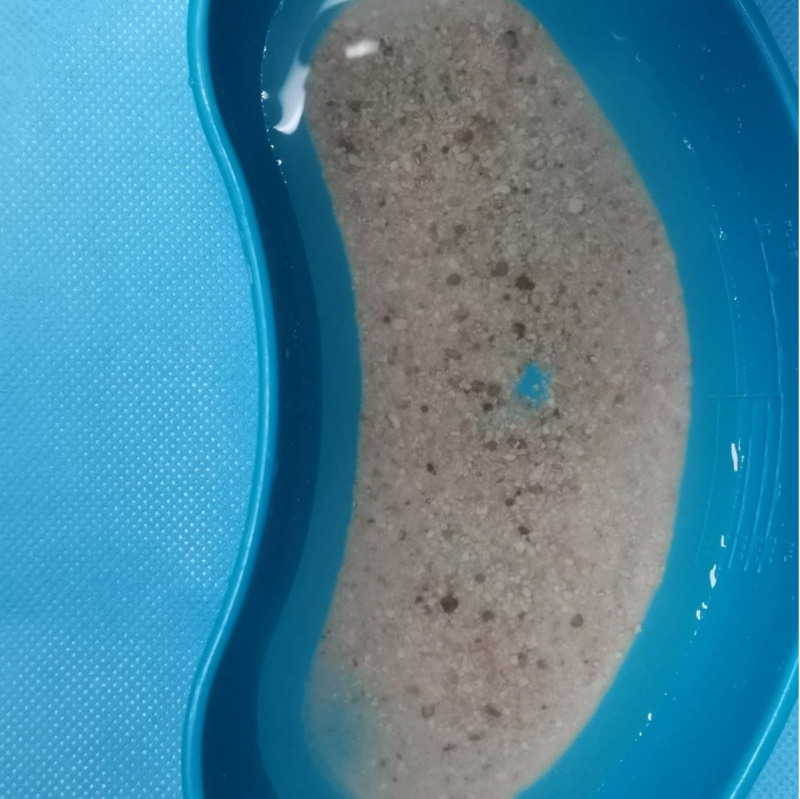
Intraoperative image of the calculus from the second surgery.

**Figure 5.**
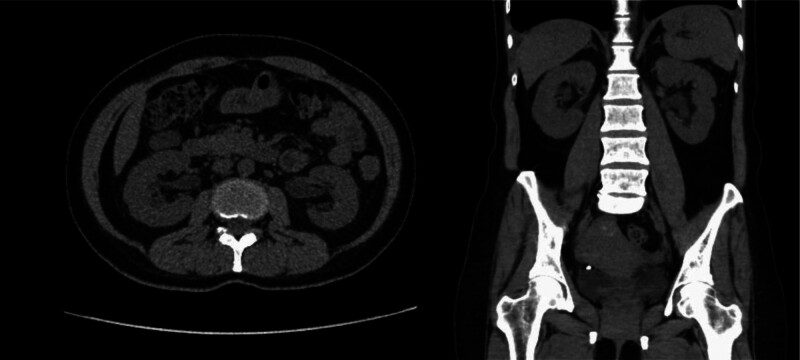
Postoperative computed tomography images showed no significant residual stones after the ureteral stent was removed.

Under general anesthesia, the patient was placed in the lithotomy position. A 6.3 Fr disposable flexible ureteroscope was inserted into the bladder through the urethral meatus, under direct vision. A right ureteral double-J stent was placed. The double-J stent was grasped with foreign body forceps and removed, and a guidewire was inserted into the renal pelvis through the stent pathway. The subsequent procedure was identical to that used during the first surgery. After clearing the remaining right kidney stone fragments, the stones in the left kidney were managed following the same steps (Fig. 4). An F5 Marflow double-J stent was placed in both ureters and an F16 Foley catheter was inserted. Anesthesia was satisfactory, and the procedure was completed successfully, with an estimated intraoperative blood loss of approximately 2 mL. The first day after the second surgery, the patient’s white blood cell was 13.19 × 10^9^/L, interleukin-6 was 29.2 pg/mL, C-reactive protein was 13.4 mg/L, and kidney function showed no significant abnormalities. The patient had an uneventful postoperative recovery. The Foley catheter was removed on the first postoperative day, and the patient was discharged the following day. A follow-up CT scan 2 weeks after surgery confirmed complete stone clearance, and the bilateral ureteral stents were removed without complications (Fig. 5). Postoperative stone composition analysis revealed the presence of carbonate apatite and magnesium ammonium phosphate hexahydrate.

### 2.4. Highlight box

- Smaller sheath diameter: The 6.3 Fr sheath of the flexible ureteroscope causes less trauma to the ureter and smaller renal calyces, reduces the indwelling time of the ureteral stent, and reduces postoperative discomfort.
- Improved irrigation flow and lower intrarenal pressure: A smaller scope diameter reduces the sheath-to-scope ratio, enhances irrigation efficiency, and improves the clearance of laser-generated stone fragments. Simultaneously, it maintains low intrarenal pressure, which is critical for preventing pyelovenous backflow and infection, thereby reducing the risk of postoperative infections. This is especially important for the management of complex stones.
- Increased success rate of single-stage surgery: The high maneuverability and efficiency of the 6.3 Fr flexible ureteroscope allow for more thorough stone fragmentation and clearance, significantly improving the success rate of a single surgical procedure.

## 3. Discussion

Staghorn calculi are among the most challenging conditions in the field of urology, with treatment goals focused on achieving complete stone clearance, preventing recurrence, and minimizing complications.^[2]^ Current standard treatment options include PCNL, combination therapy (PCNL and fURS), and, in rare cases, open surgery. PCNL is considered the gold standard for managing large-volume stones and is capable of clearing most stones in a single procedure.^[8]^ However, PCNL is highly invasive, requires a longer hospital stay, and carries the risk of serious complications, such as renal parenchymal damage, bleeding, and infection. It is contraindicated in patients with bleeding tendencies or in long-term anticoagulation therapy.^[5]^ Therefore, for patients in whom PCNL poses significant risks, or for those seeking less invasive but effective treatment for large stones, fURS serves as an ideal alternative.^[9]^

**Figure 6.**
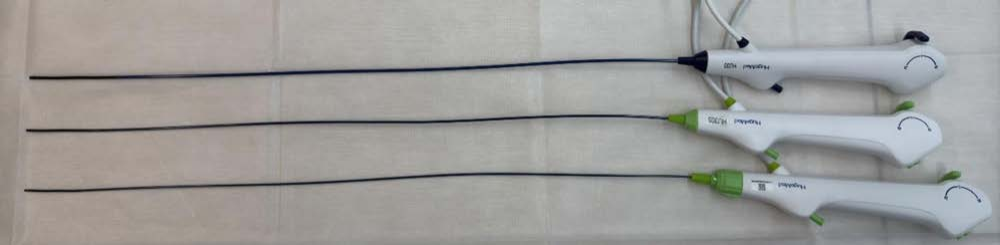
These are 3 different diameters of HugeMed disposable digital flexible ureteroscopes, measuring 9.0, 7.5, and 6.3 Fr (from top to bottom), with similar handle and port designs.

**Figure 7.**
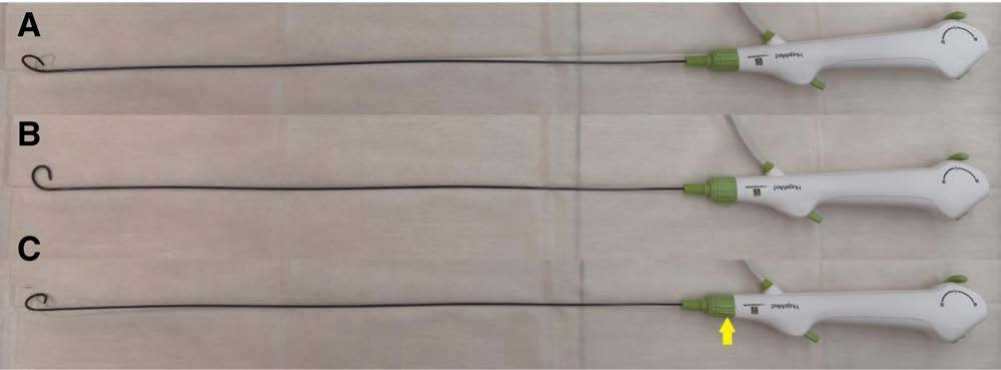
The 6.3 Fr ureteroscope is equipped with a coaxial rotation function. In the image, the yellow arrow indicates the operation point for left and right rotation of the axis, with a maximum bilateral rotation of 120°. (A) Rotation to the left. (B) Neutral position. (C) Rotation to the right.

With advances in technology, the advent of small-diameter flexible ureteroscopes has provided an innovative and minimally invasive solution for managing complex stones. In our case, a new 6.3 Fr disposable digital flexible ureteroscope was successfully used to treat bilateral staghorn calculi in a patient through 2 surgical procedures. The ureteroscope used in this case is currently the thinnest commercially available disposable flexible ureteroscope (Fig. 6). While the shaft diameter was maintained at 6.3 Fr, its working channel was equivalent to that of conventional 7.5 Fr or larger ureteroscopes, measuring 3.6 Fr. It also features a 285° bidirectional deflection capability, allowing the insertion tube to rotate by 120° to either side via a control knob (Fig. 7). Therefore, its visual performance and operational capabilities are comparable with those of the larger flexible ureteroscopes. The smaller sheath diameter of the 6.3 Fr ureteroscope significantly reduces mechanical trauma to the ureter compared with the 7.5 Fr ureteroscope, minimizing the risk of postoperative ureteral stricture, inflammation, and discomfort, while also avoiding obstructions caused by trauma during instrument passage. Additionally, unlike PCNL, it does not require the establishment of a tract through the renal parenchyma, further reducing the damage to the renal tissue.

**Figure 8.**
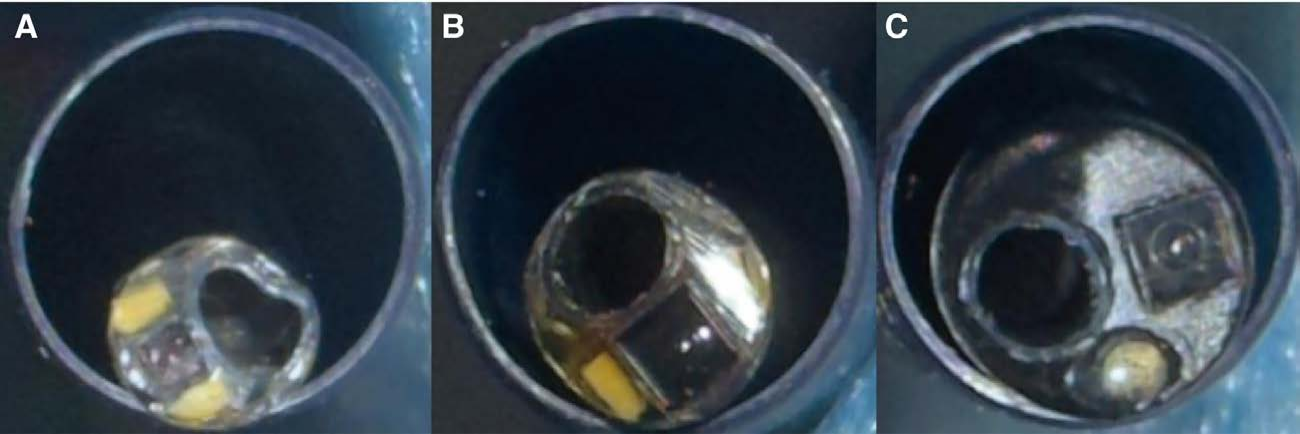
Cross-sectional comparison of 3 different diameters of flexible ureteroscope in ureteral sheaths of the same diameter. (A) A 6.3 Fr flexible ureteroscope with a 12 Fr ureteral sheath. (B) A 7.5 Fr flexible ureteroscope with a 12 Fr ureteral sheath. (C) A 9.0 Fr flexible ureteroscope with a 12 Fr ureteral sheath.

Studies have shown that when intrapelvic pressure exceeds 40 cmH_2_O, it may lead to pyelosinus, pyelovenous, and pyelolymphatic backflow, which can further cause morphological and physiological changes in the kidney and increase the risk of complications such as infection, sepsis, subcapsular hematoma, and perirenal hematoma, highlighting the critical importance of maintaining low intrapelvic pressure, particularly when managing larger intrarenal stones.^[10–12]^ The ratio between the diameter of the ureteroscope and ureteral access sheath (RESD [ratio of endoscope-to-sheath diameter]) is a key determinant of intrapelvic pressure. Studies have shown that when RESD ≥0.87, intrapelvic pressure can exceed 40 cmH_2_O even with an irrigation pressure of only 250 cmH_2_O. Conversely, when RESD ≤0.75, intrapelvic pressure remains below 13 cmH_2_O even with irrigation pressures as high as 500 cmH_2_O.^[13]^ In vitro studies have also recommended maintaining an RESD of less than 0.85.^[14]^ Furthermore, a lower RESD not only significantly reduces intrapelvic pressure but also ensures higher irrigation flow rates, providing a clear surgical field.^[13]^ The 6.3 Fr flexible ureteroscope offers an optimized endoscope-to-sheath ratio. When paired with a 12/14 Fr ureteral access sheath, the RESD was 0.525, and when paired with a 10/12 Fr sheath, the RESD is 0.63 (Fig. 8). This smaller endoscope-to-sheath ratio optimizes irrigation flow and helps maintain low intrarenal pressure during procedures. This design feature significantly reduces the likelihood of pyelovenous backflow and bacterial entry into the bloodstream, thereby lowering the risk of postoperative infection. Maintaining a low-pressure environment is particularly critical when managing staghorn calculi as it helps minimize the occurrence of systemic infections and sepsis.

The smaller diameter of the 6.3 Fr ureteroscope provides greater operational flexibility. Additionally, the ureteroscope can achieve a 120° coaxial rotation, allowing it to access complex renal calyceal structures, particularly distal areas that are difficult to reach with traditional instruments. A smaller scope diameter ensures smoother irrigation, which helps to reduce the temperature increase caused by high-power laser lithotripsy. Simultaneously, it provides a larger channel for negative pressure stone fragment evacuation, improving the efficiency of stone fragment removal and reducing the risk of postoperative steinstrasse formation. The smaller diameter also holds significant potential for future applications in pediatric urolithiasis management, as the average maximum inner diameter of the pediatric ureter is approximately 3.8 mm, which imposes specific requirements on the instruments used.^[15]^

**Figure 9.**
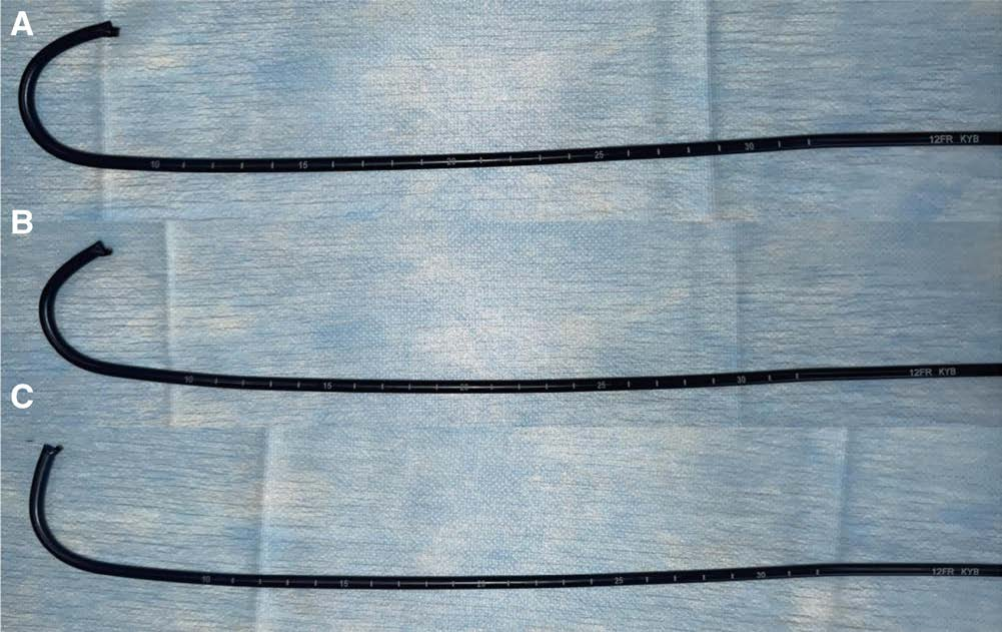
Three flexible ureteroscopes with different diameters caused the sheath of the same diameter to bend passively at different angles. (A) A 9.0 Fr flexible ureteroscope with a 12 Fr ureteral sheath. (B) A 7.5 Fr flexible ureteroscope with a 12 Fr ureteral sheath. (C) A 6.3 Fr flexible ureteroscope with a 12 Fr ureteral sheath. The smaller diameter flexible ureteroscope had a weaker ability to passively bend the sheath.

Although the results of this case are encouraging, the 6.3 Fr flexible ureteroscope has certain limitations. Compared with PCNL, the main advantages of the 6.3 Fr ureteroscope are its minimally invasive nature, flexibility, and more patient-friendly recovery process. However, its stone fragmentation and clearance times are relatively longer, and it may be less efficient than PCNL for extremely large or high-density stones. Additionally, prolonged operation time may increase operator fatigue in high-complexity cases. Owing to its thinner shaft, the 6.3 Fr ureteroscope has a weaker ability to passively bend the sheath than thicker ureteroscopes, and its thinner structure also increases the likelihood of damage to the scope (Fig. 9). These issues warrant further exploration in the design of future ureteroscopes. Future studies should focus on validating the effectiveness of the 6.3 Fr ureteroscope across different stone types and complex cases, optimizing surgical strategies, and exploring more efficient combination treatment approaches.

## 4. Conclusion

The 6.3 Fr flexible ureteroscope demonstrated unique clinical advantages in the treatment of bilateral staghorn calculi, including reduced intraoperative trauma, lower intrarenal pressure to minimize the risk of postoperative infection, improved stone clearance efficiency, and increased single-session success rate. The success of this case indicates a promising future for this technology in minimally invasive urology. However, its limitations highlight the need for further research and improvements. Future studies should involve larger sample sizes of urinary stone cases, including both pediatric and adult patients, to promote broader application in complex cases.

## Acknowledgments

The authors thank the patient for giving them the permission to use the medical information for this publication.

## Author contributions

**Conceptualization:** Feihong Xu, Zhenquan Lu, Lin Xiong, Xiang Xu.

**Data curation:** Feihong Xu, Rongfang Zhong, Weiyong Zhong, Shujiang Ye.

**Writing—original draft:** Feihong Xu.

**Writing—review & editing:** Feihong Xu, Rongfang Zhong, Weiyong Zhong, Shujiang Ye, Zhenquan Lu, Lin Xiong, Xiang Xu.
